# Supplementary material for: Using Machine Learning to Develop and Validate an In-Hospital Mortality Prediction Model for Patients with Suspected Sepsis
Source: Biomedicines. 2022 Mar 29;10(4):802. doi: 10.3390/biomedicines10040802 (PMC9030924; doi:10.3390/biomedicines10040802)
Supplement: Supplementary file 1 [file biomedicines-10-00802-s001.zip › biomedicines-1622076-supplementary.pdf]

Table S1. Relevant studies about 28-day mortality prediction for sepsis patients.

Authors	Title	Dataset	Methodology	Predictors	Outcome	Sepsis	AUROC
Masson, S. et al.	Presepsin (soluble CD14 subtype) and procalcitonin levels for mortality prediction in sepsis: data from the Albumin Italian Outcome Sepsis trial	A multicentre, randomised Albumin Italian Outcome Sepsis trial, 100 patients	Cox regression model	Presepsin level, procalcitonin level and some covariates	28-day/ICU/90-day mortality	Sepsis-2	
Fang W-F et al.	Development and validation of immune dysfunction score to predict 28-day mortality of sepsis patients	Sepsis patients admitted to ICU at a hospital in Taiwan, 151 patients	LR	Monocyte HLA-DR* expression, plasma G-CSF* level, plasma IL*-10 level, and serum SeMo* ratio	28-day mortality	Sepsis-3	0.85 (0.75-0.94)
Jau-Woei Perng et al.	Mortality Prediction of Septic Patients in the Emergency Department Based on Machine Learning	Sepsis patients admitted to ICU at a hospital in Taiwan, 42,220 patients	RF, KNN, SVM, SoftMax	Clinical variables (53) demographic data, vital signs, and laboratory results	72-hours/28-day in-hospital mortality	Sepsis-2	72h: RF(0.88) SVM (0.93) KNN(0.83) SoftMax(0.91) 24d: RF(0.79) SVM(0.93) KNN(0.84) SoftMax(0.93)
Nianzong Hou et al.	Predicting 30-days mortality for MIMIC-III patients with sepsis-3: a machine learning approach using XGboost	MIMIC-III, 4,559 patients	LR, SAPS-II score, XGBoost	demographics, vital signs, laboratory tests, fluid balance and vital status	30-day mortality	Sepsis-3	LR 0.82 (0.8-0.84) SAPS-II 0.80 (0.78-0.81) XGBoost 0.86 (0.84-0.88)

Abbreviations: *ICU* Intensive Care Unit, *LR* Logistic regression, *RF* Random forest, *KNN* K-nearest neighbor, *SVM* Support vector machine

Table S2. Biomarker distribution of the different subgroups.

Biomarkers (Median(IQR))	Total	Sepsis-1[Ŧ] (SIRS score≥2)	Sepsis-3[ŦŦ] (ΔSOFA≥2)	Septic Shock (ΔSOFA≥2+ Lactate>18 mg/dL)	Septic Shock (ΔSOFA≥2+ Lactate>18 mg/dL)+ Vasopressor usage
**Numbers (%)**	555 (100)	418 (75.32)	101 (18.20)	58 (10.45)	7 (1.26)
**Procalcitonin (ng/mL)**	0.59 (0.1-5.98)	0.79 (0.13-7.13)**	3.27 (0.73-20.06)***	6.01 (1.06-79.72)***	200 (28.90-200)***
**Lactate (mg/dL)**	14.7 (10.7-22.20)	15.3 (10.9-23.35)*	20 (13.23-30.05)***	28.4 (22.30-39.80)***	36.5 (24.9-68.65)***
**C reactive protein (mg/L)**	88.5 (40.60-160.94)	94 (37.84-164.72)	121.1 (59.60-172.5)**	115.52 (57.92-196.93)*	212.06 (138.72-249.95)**
**D-dimer (ng/mL)**	1295 (556.75-2858)	1370 (605.5-3054)**	2168 (1103-6673.75)***	2681 (1452-10000)***	6149 (2367.5-10000)**
**IL-6 (pg/mL)**	78.71 (41.79-148.76)	82.63 (42.28-161.97)*	114.46 (46.28-227.83)**	159.08 (56.37-253.25)**	175.33 (106.92-243.75)
**IL-8 (pg/mL)**	5.29 (2.61-13.49)	5.70 (2.79-14)	6.95 (3.33-14.00)	11.32 (4.25-19.47)**	14 (10.54-26.14)
**IL-10 (pg/mL)**	0.95 (0.10-3.25)	1.20 (0.14-3.55)**	1.15 (0.31-3.54)	1.89 (0.38-5.95)**	6.32 (3.34-8.78)*
**TNF-α (pg/mL)**	22.94 (14.7-35.30)	22.59 (14.67-34.46)	23.48 (17.08-34.53)	23.79 (17.63-38.5)	33.05 (27.-39.09)
**IFN-γ (pg/mL)**	0.065 (0.065-0.12)	0.065 (0.065-0.12)	0.065 (0.065-0.2)***	0.09 (0.065-0.2)***	0.13 (0.065-0.2)
**Angiopoetin-2 (pg/mL)**	827.01 (404.66-1510.85)	842.93 (408.63-1571.53)	1219.78 (645.88-1723.31)*	1258.62 (653.05-1805.59)*	878.65 (878.65-878.65)
**Pantraxin-3 (ng/mL)**	1.20 (0.60-2.29)	1.30 (0.65-2.51)***	1.30 (0.71-2.49)	1.91 (0.96-3.34)***	2.14 (1.53-2.72)
**sCD14 (ug/mL)**	2.27 (1.86-2.70)	2.21 (1.84-2.68)	2.43 (1.99-2.85)**	2.37 (1.98-2.88)	2.39 (1.87-3.38)
**sCD64 (ng/mL)**	86.55 (47.93-133.73)	93.15 (53.08-136.95)**	119.57 (80.74-180.49)***	151.39 (81.92-187.98)***	145.44 (98.22-189.28)
**sCD-163 (ng/mL)**	150.94 (104.26-251.28)	150.99 (104.26-243.60)	172.99 (110.45-257.13)	190.16 (146.04-291.24)	275.26 (257.13-310.06)
**Triggering receptor expressed on myeloid cells 1 (pg/mL)**	276.72 (9.49-514.32)	295.09 (9.49-538.20)	334.26 (234.12-492.09)	367.46 (241.38-564.07)	793.18 (638.27-849.46)*
**Intercellular Adhesion Molecule 1 (ng/mL)**	178.69 (103.61-277.12)	184.32 (109.84-292.34)**	226.30 (137.69-343.71)**	256.81 (142.45-339.68)*	244.1 (174.96-329.58)
**Vascular cell adhesion protein 1 (ng/mL)**	1.76 (1.17-2.65)	1.76 (1.25-2.59)	2.48 (1.49-3.60)***	2.68 (1.54-3.94)***	4.19 (3.51-4.62)**
**E-selectin (ng/mL)**	57.09 (37.01-101.49)	62.78 (39.28-106.85)**	70.11 (45.60-115.98)*	78.63 (43.21-116.17)	78.63 (51.05-150.80)
**P-selectin (ng/mL)**	52.34 (38.74-66.03)	54.39 (40.15-67.43)***	49.85 (38.95-69.57)	59.56 (39.21-70.62)	65.61 (52.70-75.62)
**Procalcitonin (ng/mL)**	0.59 (0.1-5.98)	0.79 (0.13-7.13)**	3.27 (0.73-20.06)***	6.01 (1.06-79.72)***	200 (28.90-200)***

Note: * $P < 0.05$, ** $P < 0.01$, and *** P-value < 0.001 represent the difference between Sepsis-1, Sepsis-3, and septic shock compared to the corresponding control group.

Table S3. Comparison of level of sepsis-related novel biomarkers between the survivors and the in-hospital mortality groups.

Biomarkers Median (IQR)	Survivor(n=510)	Death(n=45)	*p*-value
**IL-6 (pg/mL)**	76.09 (41.71-147.08)	116.83 (47.44-265.48)	0.084
**IL-8 (pg/mL)**	5.07 (2.43-12.14)	13.65 (5.94-17.52)	<0.001
**IL10 (pg/mL)**	0.93 (0.10-3.08)	2.04 (0.71-5.04)	0.002
**Angiopoetin-2 (pg/mL)**	814.70 (407.61-1472.72)	1216.00 (371.96-1938.29)	0.258
**Pantraxin (ng/mL)**	1.18 (0.59-2.20)	1.57 (1.05-2.68)	0.019
**sCD14 (ng/mL)**	2.25 (1.86-2.68)	2.35 (1.85-2.85)	0.507
**sCD64 (ng/mL)**	84.60 (47.85-132.33)	96.92 (71.94-164.77)	0.061
**sCD163 (ng/mL)**	148.13 (103.56-236.43)	227.10 (136.54-324.85)	0.002
**TREM1 (pg/mL)**	267.16 (6.99-495.36)	452.8 (230.76-880.05)	0.001
**ICAM1(ng/mL)**	165.13 (100.56-268.18)	226.38 (181.11-355.87)	0.001
**VCAM1 (ug/mL)**	1.74 (1.16-2.55)	2.32 (1.56-3.00)	0.011
**E-selectin (ng/mL)**	56.99 (36.54-101.12)	60.81 (45-103.41)	0.387
**P-selectin (ngr/mL)**	51.43 (38.56-64.95)	61.19 (45.02-77.02)	0.034

TNF-α: tumor necrosis factor-alpha, IL-6: interleukin-6, IL-8: interleukin-8, IL-10: interleukin-10 and IFN-γ: interferon-gamma, sCD14: soluble cluster of differentiation-14, sCD64: soluble cluster of differentiation-64, sCD163: soluble cluster of differentiation-163,TREM-1: triggering receptor expressed on myeloid cells-1, ICAM-1: intercellular adhesion molecule-1 and VCAM-1: vascular cell adhesion protein-1.

Table S4. Total included features.

Number	Features
**1**	Age
**2**	temperature
**3**	Pulse centering value of 100
**4**	Systolic blood pressure centering value of 120
**5**	Diastolic blood pressure centering value of 75
**6**	Respiratory rate centering value of 18
**7**	oxyhemoglobin saturation by pulse oximetry
**8**	Mean arterial pressure centering value of 75
**9**	Glasgow Coma Scale

**10**	Angiopoietin-2 normalization
**11**	IL-6 normalization
**12**	TNF-a normalization
**13**	sCD163 normalization
**14**	IL-10 normalization
**15**	Pentraxin-3 normalization
**16**	normalization
**17**	normalization
**18**	CD14 normalization
**19**	TREM-1 normalization
**20**	CD64 normalization
**21**	ICAM-1 normalization
**22**	E-selectin normalization
**23**	P-selectin normalization
**24**	VCAM-1 normalization
**25**	Fasting Blood Glucose centering value of 180
**26**	Procalcitonin
**27**	C Reactive Protein
**28**	Lactate
**29**	Albumin
**30**	APTT centering value of 30
**31**	Prothrombin
**32**	INR
**33**	Bilirubin
**34**	BUN
**35**	AST
**36**	Phosphorous centering value of 3
**37**	Troponin-I
**38**	D-dimer
**39**	Cortisol
**40**	Calcium centering value of 8.75
**41**	Chloride
**42**	Uric Acid
**43**	C3
**44**	Protein C centering value of 75
**45**	pO2
**46**	SAT
**47**	HCO3 centering value of 25
**48**	pCO2
**49**	pH
**50**	Total CO2
**51**	ABE
**52**	AaDO2
**53**	FiO2
**54**	SBC
**55**	SBE
**56**	FDP
**57**	BAND
**58**	Serum Creatinine
**59**	Eosinophils
**60**	Hemoglobin
**61**	Hematocrit
**62**	MCHC
**63**	MCV
**64**	Platelet
**65**	Red Blood Cell
**66**	RDW

**67**	White Blood Cell centering value of 15
**68**	Potassium
**69**	Sodium
**70**	high blood pressure
**71**	Asthma
**72**	Gout
**73**	Parkinson
**74**	HIV
**75**	Stroke
**76**	Allergy
**77**	myocardial infarction
**78**	Congestive Heart Failure
**79**	Peripheral Vascular Disease
**80**	Cerebrovascular Disease
**81**	Dementia
**82**	Chronic Pulmonary Disease
**83**	Rheumatologic Disease
**84**	Peptic Ulcer Disease
**85**	Mild Liver Disease
**86**	Hemiplegia or paraplegia
**87**	Renal Disease
**88**	Leukemia
**89**	Lymphoma
**90**	Moderate or Severe Liver Disease
**91**	Metastatic Solid Tumor
**92**	Tumor
**93**	Chronic Kidney Disease
**94**	Carrier
**95**	Fatty Liver
**96**	Cirrhosis Liver
**97**	Liver Disease
**98**	Chronic Obstructive Pulmonary Disease
**99**	Any Malignancy
**100**	Diabetes mellitus
**101**	Respiratory infection
**102**	Urology infection
**103**	Skin infection
**104**	Abdominal infection
**105**	Central nervous system
**106**	Musculoskeletal infection
**107**	Other infection
**108**	Cardiovascular dysfunction
**109**	Respiratory dysfunction
**110**	Gastrointestinal tract
**111**	Renal dysfunction
**112**	Hepatic dysfunction
**113**	Neurologic dysfunction
**114**	Metabolic dysfunction
**115**	Hematologic dysfunction
**116**	Sequential Organ Failure Assessment (SOFA) Score
**117**	Sepsis-3, SOFA Score>=2
**118**	Septic shock , SOFA Score>=2>=2 and Lactate>18
**119**	Body aches
**120**	Muscle ache
**121**	Convulsion
**122**	General Weak
**123**	Chills

**124**	Shaking Chill
**125**	Cyanosis
**126**	Fever
**127**	Sweat
**128**	No sweat
**129**	Dry Lips
**130**	Thirsty
**131**	Cold extremity
**132**	Malaise
**133**	Drowsy
**134**	Syncope
**135**	Confusion
**136**	Tachycardia
**137**	Agitation
**138**	acute confuse
**139**	Fluctuating confuse
**140**	Inattention
**141**	Disorganized think
**142**	Urine frequency
**143**	Oliguria
**144**	Hematuria
**145**	Difficulty urinating
**146**	Dysuria
**147**	Flan pain
**148**	Headache
**149**	Dizziness
**150**	Sore throat
**151**	Chest tightness
**152**	Dyspnea
**153**	Chest pain
**154**	Neck pain
**155**	Nasal congestion
**156**	Sneezing
**157**	Runny nose
**158**	Dry cough
**159**	Productive cough
**160**	Sputum
**161**	Hemoptysis
**162**	Abdominal diet
**163**	Abdominal pain
**164**	Diarrhea
**165**	Constipation
**166**	Nausea
**167**	Vomit
**168**	Anorexia
**169**	Jaundice
**170**	Hypotension
**171**	lactate higher
**172**	Creatinine over 2
**173**	Bilirubin over 2
**174**	Platelet less than 100
**175**	INR over 15
**176**	Male
**177**	an infiltrate on chest imaging
**178**	chest X-ray with consolidation from pneumonia
**179**	pneumonia patch
**180**	pulmonary edema

**181**	hazy shadow on chest x ray
**182**	Pneumonia
**183**	Upper respiratory tract infection
**184**	Patient older than 65 years old
**185**	Lower respiratory infection
**186**	Patient is from a nursing home
**187**	Mental status is altered
**188**	Granulocytic bands >5% of WBC
**189**	Platelet count below 150,000
**190**	Shock from sepsis
**191**	Hypoxia or tachypnea
**192**	Terminal illness with possible death in 1 month
**193**	Mortality in emergency department sepsis score
**194**	Temperature >38°C or < 36°C
**195**	Heart rate > 90
**196**	Respiratory rate > 20
**197**	WBC > 12,000/mm^3 or < 4,000/mm^3
**198**	SIRS score
**199**	Altered Mental Status
**200**	Dopamine <5 or dobutamine (any dose)
**201**	Dopamine 5.1-15 or epinephrine ≤0.1 or norepinephrine ≤0.1b
**202**	Dopamine >15 or epinephrine >0.1 or norepinephrine >0.1b
**203**	Respiratory System
**204**	Nervous System
**205**	Cardio Vascular System
**206**	Liver System
**207**	Coagulation System
**208**	Renal System
**209**	Vasopressor
**210**	Bacteremia
**211**	Intensive care unit
**212**	Severe sepsis
**213**	Chills
**214**	Hypothermia (temperature< 36°C)
**215**	Red blood cell volume distribution width >14.5%
**216**	Anemia (RBC counts < 4 million/μL)
**217**	No complaints of chill
**218**	novel clinical prediction rule
**219**	No blood drawn

Table S5. The corresponding mean weighted contribution and area under the receiver operating characteristic curves (AUROC) of 30 features selected by the wrapper algorithm around the random forest models in the training dataset. The feature candidates were ranked according to the AUROC.

Features	[δ]Mean weighted contribution	AUROC
**IL-8**[*]	**9.34**	**0.83**
**SOFA total score**	**12.63**	0.82
**Albumin**	**5.23**	0.80

**D-dimer**	**6.74**	0.77
**Cortisol, ug/dL**	**3.04**	0.74
**Lactate**	**3.57**	0.73
**SOFA score -Respiratory**	**5.18**	0.72
**Red Blood Cell**	**4.07**	0.71
**IL-6***	**4.99**	0.71
**FDP**	**4.50**	0.69
**SBC**	**3.88**	0.69
**Angiopoetin2**	**4.40**	0.69
**SOFA score -Coagulation**	**2.98**	0.68
**Platelet**	**6.37**	0.68
**Septic shock**	**2.76**	0.67
**Procalcitonin**	**4.19**	0.66
**Calcium, centered at 8.75 mg/dL**	**2.58**	0.65
**HCO3 centered at 25 mmol/L**	**3.46**	0.64
**Uric Acid**	**3.40**	0.64
**VCAM1***	**3.78**	0.64
**pH**	**4.99**	0.63
**Pulse rate, centered at 100 BPM**	**3.05**	0.61
**Highest SOFA score cardiovascular**	**2.74**	0.59
**AaDO2**	**5.07**	0.58
**pCO2**	**3.23**	0.58
**SBE**	**3.79**	0.56
**ABE**	**3.97**	0.56
**FiO2**	**2.95**	0.55
**Total CO2**	**4.55**	0.55
**E-selectin***	**3.86**	0.54

*Values are normalized. $^{\delta}$ The weighted contribution represents the importance and degree of influence of individual features in the dataset. Highly relevant features indicate a high probability of reaching the classification node and are given high weighted values, whereas irrelevant features weighted low values. Highest SOFA score cardiovascular: dopamine > 15 μg/kg/min OR epinephrine > 0.1 μg/kg/min OR norepinephrine > 0.1 μg/kg/min

Table S6. The AUROC performance of seven machine learning models when various features were selected by applying the SMOTE for both up-sampling down-sampling procedure.

Models	Dataset	Without SMOTE	With SMOTE(up-sampling)	With SMOTE(down-sampling)
**Random Forest**	Training	1.00 (1.00-1.00)	1.00 (1.00-1.00)	1.00 (1.00-1.00)
	Testing	**0.96 (0.93-0.98)**	0.92 (0.87-0.97)	0.94 (0.89-0.99)

Table S7. Sequential (sepsis-related) Organ Failure Assessment (SOFA) score.

Variable	Index	0	1	2	3	4
**SOFA score**						
**SOFA_res**	PaO2/FiO2, mmHg (kPa)	>=400 (53.3)	<400 (53.3)	<300 (40)	<200 (26.7) with respiratory support	<100 (13.3) with respiratory support
**SOFA_coag**	Platelets, $x10^3$/uL	>=150	<150	<100	<50	<20
**SOFA_liver**	Bilirubin, mg/dL (umol/L)	<1.2 (20)	1.2-1.9 (20-32)	2.0-5.9 (33-101)	6.0-11.9 (102-204)	>12.0 (204)
**SOFA_vas**	MAP, Dop	MAP >= 70 mmHg	MAP < 70 mmHg	Dopamine <5 (ug/kg/min) or Dobutamine (ug/kg/min)	Dopamine 5.1-15 (ug/kg/min) or Epinephrine <= 0.1 or Norepinephrine <= 0.1	Dopamine >15 (ug/kg/min) or Epinephrine > 0.1 or Norepinephrine > 0.1
**SOFA_renal**	Creatinine, mg/dL (umol/L)<br>Urine Output, mL/d	<1.2 (110)	1.2-1.9 (110-170)	2.0-3.4 (171-299)	3.5-4.9 (300-440)<br><500	>5.0 (440)<br><200
**SOFA_ner**	Glasgow coma score	>=15	13-14	10-12	6-9	<6
**Sepsis-3**	SOFA score>=2					
**Septic shock**	SOFA score >=2 and Lactate >18 mmol/L					
**ΔSOFA score**	Difference between measured and baseline SOFA score					

Table S8. The summary descriptions of the selected eight algorithms.

Algorithms	Description
**XGBoost**	eXtreme Gradient Boosting (XGBoost) is a decision-tree-based algorithm that applies boosting methods to further improve performance on those misclassified observations.
**CForest**	Conditional random forest (CForest) is computationally more expensive and better than the Random forest package in terms of accuracy. CForest uses out-of-bag data to provide higher accuracy. It then uses a weighted average of the trees to get the final ensemble. CForest provides more reliable predictions by producing unbiased trees.
**Random Forest**	RF forms bagged decision tree models, which split on a subset of features on each split. By creating a multitude of decision trees to be trained on the training dataset, the predictive output value is the mode of the classes (classification) or mean prediction (regression) of the individual tree. By averaging away the variances of a number of trees, it helps to reduce the high variance derived from a single tree.
**RANGER**	RANdom forest GEneRator could build models quickly and find out optimal parameter values using parameter tuning. RANGER is used when dealing with high dimensional data and expects a memory-efficient fast implementation of RF.
**ANN**	Artificial Neural Networks are complex and flexible nonlinear systems. Hidden nodes link features with the outcomes, allowing nonlinear interactions among the features.
**SVM**	A support-vector machine constructs hyperplanes in a high- or infinite-dimensional space, which can be used for classification, regression, or other tasks like outliers detection. When the datasets to be discriminated are not linearly separable in a finite-dimensional space, SVM helps mapping into a much higher-dimensional space, making the separation and classification easier in that space.
**Deep Learning**	The deep learning method makes use of artificial neural networks, which constructs multiple layers to progressively extract higher-level features from the raw input. For supervised learning tasks, it translates the data into compact, intermediate representations akin to a principal component, and derives layered structures that remove redundant engineering features, thus can serve as validation of other machine learning algorithms.
**Logistic regression**	Logistic regression is similar to linear regression, which discovered the relationship between independent features (X) and dependent features (Y). The major difference is that the dependent feature (Y) is usually a continuous feature in linear regression but mainly a category feature in logistic regression.

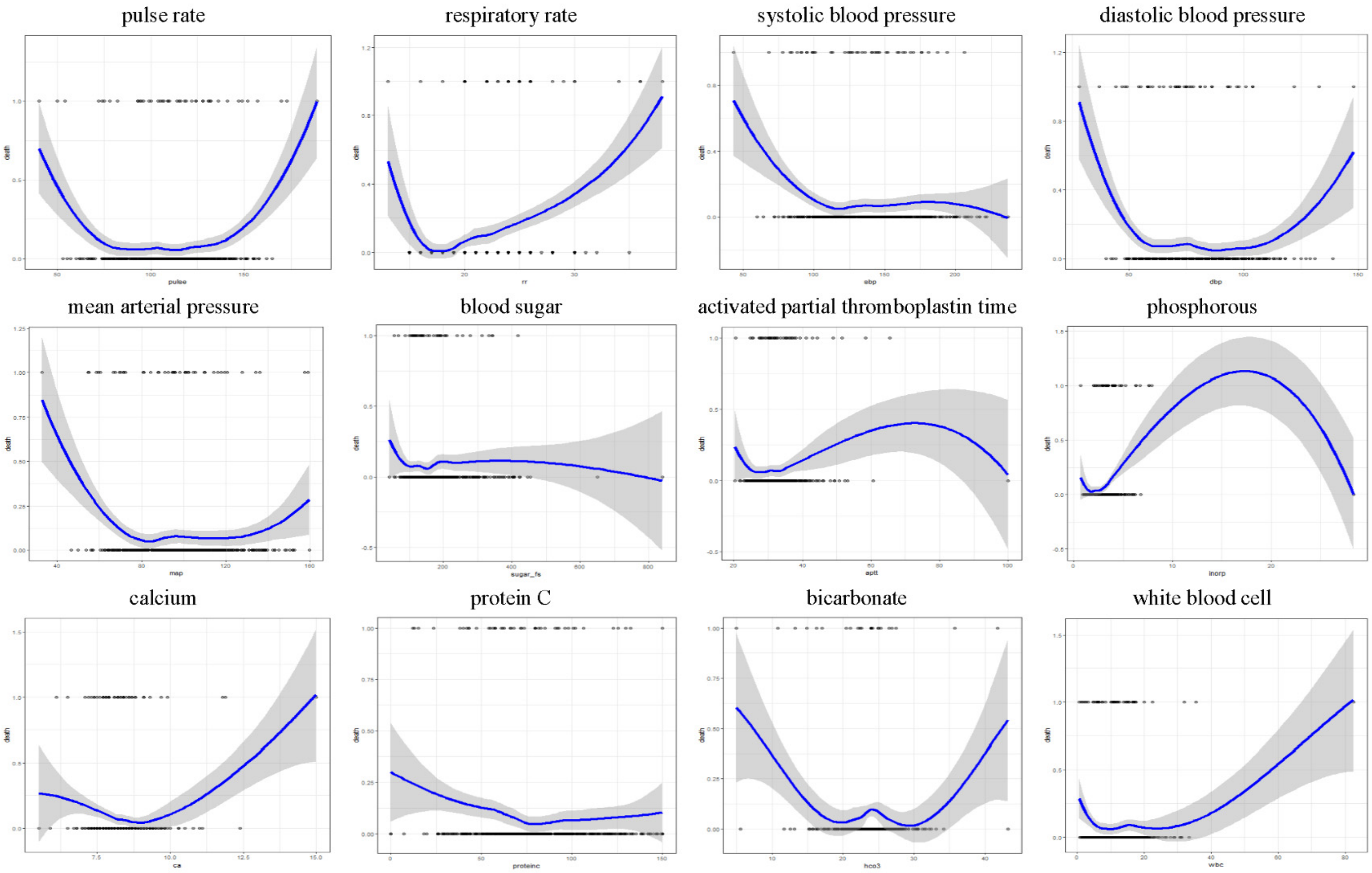

Figure S1. The U-shape distributions of 12 features.
